# Person-centred care compared with standardized care for patients undergoing total hip arthroplasty—a quasi-experimental study

**DOI:** 10.1186/s13018-014-0095-2

**Published:** 2014-10-09

**Authors:** Lars-Eric Olsson, Jón Karlsson, Urban Berg, Johan Kärrholm, Elisabeth Hansson

**Affiliations:** Institute of Health and Care Sciences, Sahlgrenska Academy, University of Gothenburg, Box 457, SE 405 30 Gothenburg, Sweden; Centre for Person-Centred Care, Sahlgrenska Academy, University of Gothenburg, Gothenburg, Sweden; Department of Orthopaedics, Institute of Clinical Sciences, Sahlgrenska Academy, University of Gothenburg, Gothenburg, Sweden; Department of Orthopaedics, Sahlgrenska University Hospital, Gothenburg, Sweden; Department of Surgery and Orthopaedics, Kungälv Hospital, Kungälv, Sweden

**Keywords:** Person-centred care, Person-centered care, General self-efficacy, Tampa scale of Kinesiophobia, ASA classification

## Abstract

**Background:**

A common approach to decrease length of stay has been to standardize patient care, for example, by implementing clinical care pathways or creating fast-track organizations. In a recent national report, it was found that Sweden’s healthcare system often fails to anticipate and respond to patients as individuals with particular needs, values and preferences. We compared a standardized care approach to one of person-centred care for patients undergoing total hip replacement surgery.

**Methods:**

A control group (*n* =138) was consecutively recruited between 20th September 2010 and 1st March 2011 and an intervention group (*n* =128) between 12th December 2011 and 12th November 2012, both scheduled for total hip replacement. The primary outcome measures were length of stay and physical function at both discharge and 3 months later.

**Results:**

The mean length of stay in the control group was 7 days (SD 5.0) compared to 5.3 days in the intervention group (SD 2.2). Physical functional performance, as assessed using activities of daily living, was similar at baseline for both groups. At discharge, 84% in the control group had regained activities of daily living level A vs. 72% in the intervention group. At 3 months after surgery, 88% in the control group had regained their independence vs. 92.5% in the person-centred care group.

**Conclusions:**

Focusing attention on patients as people and including them as partners in healthcare decision-making can result in shorter length of stay. The present study shows that the patients should be the focus and they should be involved as partners.

## Introduction

Almost 16,000 total hip arthroplasties (THA) are performed annually in Sweden, in most cases with good clinical outcomes [1]. The goal of total hip replacement is optimal pain relief and an essentially normalized health-related quality of life (HR-QoL). The time that patients spend in hospital care, or length of stay (LoS), for THA has decreased during the last few decades throughout the world [2].

The most common approach to decreasing length of stay has been to standardize patient care, for example, by implementing clinical care pathways or developing fast-track organizations [2]. However, results from the Swedish Hip Replacement Registry, including 16, 000 patients, show that approximately 14% of patients were unsure of or unhappy with the final outcome [1]. In most cases, the reasons for their dissatisfaction were unclear [1]. A recent national report found that Sweden’s healthcare system often fails to anticipate and respond to patients as individuals with particular needs, values and preferences [3]. Failure to involve patients in their own healthcare can have demonstrable costs for patients, the health system and public finances.

The importance of involving the patients themselves was emphasized in an earlier study, which showed that patients were more likely to have better scores on HR-QoL dimensions up to 1 year after THA if they were involved and pleased with the admission process and the care they received [4]. A systematic review found that THA patients with lower preoperative function reached a lower level of postoperative function compared to patients who had better preoperative function, even though individuals who already have high preoperative scores may have a smaller opportunity to benefit from surgery [5]. Modest improvement may also be associated with different types of co-morbidity. One study found that co-morbidity care was poorly coordinated prior to having surgery [6]. Following joint replacement surgery, the emphasis of care was patient flow through the healthcare system according to clinical guidelines. General well-being was shown to be less than optimal; participants reported pain, fatigue, insomnia and alterations in urinary elimination as the main reasons for discomfort [6].

PCC has been advocated as a key indicator for quality of care by the World Health Organization (WHO) and by the Institute of Medicine at the US National Academy of Science [7]. There are many definitions of PCC and one of them is the Gothenburg person-centred care (gPCC) model that asserts that patients are people and should not be reduced to their disease alone, but rather that their context, experiences, goals and wishes should be taken into account [8]. gPCC means a shift away from a model in which the patient is the passive target of a medical intervention to a model where a contractual arrangement is made involving the patient and often relatives as active partners in the care process [9]. The model has been described in detail elsewhere [9]. The gPCC model has also been shown to improve outcomes such as hospital LoS and ability to achieve activities of daily living (ADL) in patients with hip fracture as well as those with heart failure [8,10]. Standardized care produces good results, but it is insufficient and should be supplemented with a holistic approach. Our aim was to compare a standardized care approach to gPCC for patients undergoing THA surgery. The hypothesis was that person-centred care would improve patients’ recovery as measured by LoS.

## Methods

**Figure 1:**
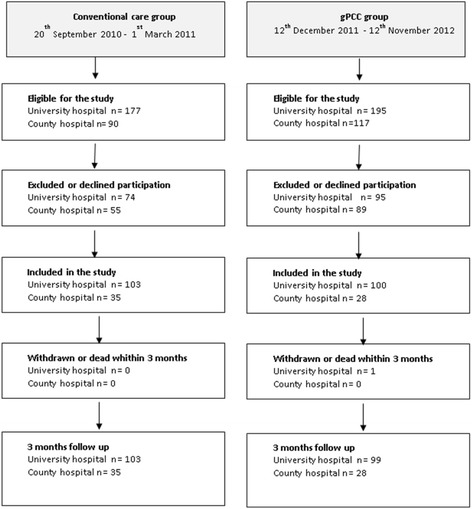
**Study flow chart.**

The study had a controlled before and after design and was carried out in three phases. Phase 1 was collecting data and mapping the control group, phase 2 was the implementation strategy and developing the gPCC and phase 3 was implementing the gPCC. A control group (*n* =138) and an intervention group (*n* =128) were consecutively recruited, and the inclusion criteria were set as scheduled for THA, able to complete instruments and willing to participate. Exclusion criteria included cognitive impairment or reluctance to participate (Figure 1).

### Conventional care

During the pre-admission examination, the patients in the control group received standardized information regarding anaesthesia, postoperative mobilization and the expected LoS. All were informed orally and also through a booklet. LoS for example was expected to be 4–5 days.

A questionnaire regarding living and health conditions was given to all the patients.

The eligible patients were treated according to the standardized routines for hip replacement surgery.

### Implementation strategy

Based on mapping the control group, an experienced multidisciplinary team that represented all the vocational groups from both departments developed measures aimed at aligning care with the basic gPCC principles [9]. The development continued from March to December 2010; altogether, 24 people participated representing the outpatient clinic and the wards. The ward group had 12 meetings, the outpatient clinics had 9 meetings and the surgeons had 7 meetings. These measures were incorporated into a study protocol to guide pre-admission, admission, treatment and discharge procedures. A reference group was invited consisting of 25 people from the “osteoarthritis” group in the local Rheumatics Association. All members of the reference group had previous experience of hospital care and some had undergone major orthopaedic surgery. The reference group discussed the relevant details of the intervention and the burden on patients of completing instruments used in the pre-admission examination. The final gPCC intervention was determined by the head of the departments. Implementation started with all hospital staff (approximately 125 people) receiving an introduction to gPCC and training on how to apply it to patient care. During the intervention period, a dedicated study nurse monitored and supported all staff in gPCC.

### gPCC intervention

The intervention aimed to give the care systematically, combine evidence-based guidelines and clinical knowledge with the patients’ individual prerequisites and form a partnership. The gPCC care was specifically designed to identify each patient’s resources and barriers and to guide the care. A comprehensive narrative was obtained from each patient, covering their everyday life, resources, motivation and goals. The patients rated themselves using the EQ-5D [11], Functional Recovery Scale (FRS) [12] and ADL [13]. After the examination, a tentative detailed healthcare plan was drawn up by a nurse built on the narrative, medical examination and self-rated surveys. This specified the patient’s short- and long-term goals, resources, special needs and plan for recovery after discharge. Based on the patient’s prerequisites, a prognosis for the LoS was also given which for most of them ranged between 2 and 7 days [14]. The tentative healthcare plan was enclosed in the letter with their appointment 2 weeks before surgery, and it was finalized when agreement had been reached. The patients were encouraged to bring a relative as support to all preoperative activities.

When the patient was admitted to the ward the day before surgery, the ward nurses had read the healthcare plan and confirmed with the patient that all facts were correct. The patients were given a checklist comprising details of the hospital care and the discharge procedure to enable involvement in their own care.

### Outcome data

The primary outcome measure was LoS, calculated as the number of whole inpatient days from admission to discharge. Secondary outcomes included physical function at both discharge and 3 months later, measured with ADL [13] and FRS [12]. ADL was self-assessed by the patients at admission and measured by a nurse at discharge. Any hospital readmission within 3 months was obtained from the patient records.

### Power analysis and statistics

A previous audit of hospital records of patients undergoing THA from the researched hospitals found that the mean length of hospital stay was 7.4. We decided to use the average LoS from the control group in the present study which was 7.01 days (SD 5.01). We estimated that 99 patients would be required in each group to achieve 80% power to detect a 2-day reduction in LoS at a significance level of *p* <0.05. Descriptive statistics were used to characterize the study groups. Between-group differences were tested using Fisher’s exact test for dichotomous variables, the Mantel–Haenszel Chi-squared test for ordered categorical variables, the Chi-squared test for non-ordered categorical variables and the Mann–Whitney *U*-test for continuous variables. The data were analysed using SPSS version 19.0 for Windows (SPSS Inc, Chicago, IL, USA).

### Ethical considerations

All patients were examined by an orthopaedic surgeon and a nurse before surgery in the outpatient clinic, where they received oral and written information about the study and provided voluntary informed consent. The Regional Ethical Review Board approved the study (Dnr: 275–10) and the investigation conforms to the principles outlined in the Declaration of Helsinki.

## Results

### Baseline characteristics

In total, 266 patients were included in the study, 138 in the control group and 128 patients in the intervention group, one lost in a follow-up. Table 1 shows baseline characteristics for the two groups. The study groups were similar except for the type of living. More patients in the control group lived in a private house (*p* =0.02) (Table 1).

**Table 1:** **Patient baseline data collected before surgery**

**Data**	**Control** ***n*** **=138**	**gPCC** ***n*** **=128**	***p*** **value**	**Data**	**Control** ***n*** **=138**	**gPCC** ***n*** **=128**	***p*** **value**
Female/male	89/49	83/45	0.9	Type of living			0.02*
Mean age	66	68	0.1	Flat	62	74	
Standard Deviation	13,9	12		House	75	52	
Living with someone	90	68	0.6	Service flat	1	2	
Living alone	46	56					
Employment status			0.2	Need of assistance from relative			1.0
Employed	32	33		Yes	71	67	
Retired	84	79		No	57	54	
Disability pension	16	5					
Other	3	6					
Contact with relatives			0.8	Need of community home help			0.7
Weekly	129	120		None	120	115	
Weekly to monthly	6	4		Once a week	6	6	
<monthly	2	2		Daily or more	7	4	
Home nursing			0.1	Assistive aids for personal use such as pincers, seat cushions and so on			0.4
Yes	1	4		Yes	46	51	
No	132	120		No	44	61	
Emergency medical alarm at home			0.7	Pre-fracture independence††			0.9
Yes	15	9		80–100%	132	117	
No	97	113		60–79%	3	7	
				<60%	3	3	
				Mean	92	92	
				SD	13	16	
Number of co-morbidities			0.06	Type of walking aid			0.5
Median	1	1		None	44	42	
Min	0	0		Crutches	64	54	
Max	6	9		Walking frame	14	19	
				Wheel chair	3	4	
ASA grade			0.1	Previous hip replacement in contralateral hip			0.4
1	36	22		Yes	47	32	
2	75	69		No	91	95	
3	27	16					
Using naturopathic preparation			1.0	Feeling healthy			0.7
Yes	116	107		Yes	78	77	
No	19	17		No	19	16	

*In this variable there was a significant difference between the groups.

†Measured by the Ceder scale [15].

†† Measured by the Functional Recovery Scale [12].

The missing data in some of the variables was regarded as not having an impact on the overall results.

*gPCC* Gothenburg person-centred care.

### Length of stay

The mean LoS in the control group was 7 days (SD 5.0) compared to 5.3 days in the gPCC group (SD 2.2) (*p* <0.0005) (Table 2). The mean LoS was longer and the standard deviations (SD) were wider for the university hospital than the county hospital in both groups (Table 3). In the control group, the expected LoS was 4–5 days, which was achieved in 54% of the patients. The LoS in the control group was prolonged between 1 and 39 days for 46% of the patients. No reason for the prolongation was documented in the medical record in 60% of patients. The reason in the remaining cases was related to nausea/vomiting/fatigue/pain (13%), leakage from the wound (7.2%), low blood count (6%) and failed social planning (7.2%) or a combination of reasons (6%). In the intervention group, the expected LoS was planned in cooperation with the patient and ranged between 3 and 7 days, which 84% achieved. A few patients in the intervention group had a prolonged stay for the same reasons.

**Table 2:** **LoS in the whole group**

	**Mean**	**SD**	**Range**	**Difference**	***p*** **value**
Control group *n* =138	7.01	5.0	2–44	1.67	<0.0005
gPCC group *n* =128	5.34	2.2	2–14

**Table 3:** **LoS in the subgroups**

		**Mean**	**SD**	**Range**	**Difference**	***p*** **value**
University hospital						
Control group	*n* =138	7.5	5.6	2–44	1.8	<0.003
gPCC group	*n* =100	5.69	2.2	3–14		
County hospital						
Control group	*n* =35	5.6	1.6	4–13	1.5	<0.001
gPCC group	*n* =28	4.1	1.6	2–8		

### Activities of daily living (ADL)

Physical functional performance, as assessed with personal ADL, was similar at baseline in the two groups: 90% were completely independent and the others needed minor assistance. At discharge, 84% in the control group had regained ADL level A compared with 72% in the intervention group, the difference was not significant. Before surgery, 3.5% in the control group scored less than 80% independency on the FRS scale compared with 6.5% in the gPCC group. Three months after surgery, 12% in the control group scored under 80% compared with 8.5% in the gPCC group and the difference was not significant.

### Health-related quality of life (EQ-5D)

The gPCC group scored lower than the controls before surgery (*p* =0.39) and slightly higher at discharge (*p* =0.44) but the differences were not significant (Table 4). The differences between pre-surgery and discharge values were significant in both groups (*p* <0.0005) (Table 4).

**Table 4:** **EQ-5D**

	**Control group**	**gPCC group**	***p*** **value (difference)**	National values*
	***n*** **=122**	***n*** **=101**		***n*** **=25,853**
Before surgery	0.359	0.313	0.393	0.41
Discharge	0.752	0.758	0.445	0.77
Gain	0.39	0.45		0.36
*p* value (gain)	*<*0.0005	*<*0.0005		

The table compares preoperative values to discharge values and between groups values measured with EQ-5D.

*Values from the Swedish Hip Arthroplasty Register are also shown [1].

### Readmission

Readmissions within 3 months were similar between the two groups; two patients in the control group and three in the gPCC group were readmitted and the difference was not significant.

## Discussion

Our main finding was that LoS was significantly reduced among patients undergoing THA who received the gPCC intervention compared to a non-concurrent control group treated according to standard clinical practice. Comparing the LoS between countries and hospitals could sometimes be misleading depending on how the preoperative time is counted. In our study, most patients were admitted to the hospital the day before surgery, adding an extra day in comparison with some studies. A change of these routines was planned but was not possible within the time frame of the study. Although the patients in the control group were aware of the planned LoS of 4–5 days, only half of them could achieve this, while in the gPCC group, most patients achieved their individually planned LoS. The reasons given in the medical records for prolonged LoS were similar for both groups, but the prolongation was less in the gPCC group. The reason for the difference was the thorough assessment of the patient as a person which resulted in a jointly developed healthcare plan. The patients in the control group were not assessed from a person-centred perspective and their individual circumstances were not as well considered as in the intervention group, so their LoS varied a lot and one patient had a stay of 44 days related to co-morbidities not addressed preoperatively. Our findings are consistent with a study of patients with co-morbidities such as diabetes, stroke, heart disease and hypertension requiring joint replacements [6]. The investigators found that co-morbidities were poorly coordinated prior to and during the hospital stay, leading to suboptimal outcomes. The patients felt that the emphasis was put on the joint surgery and patient flow through the healthcare system, leading to a neglect of their well-being [6]. Correlations between co-morbidities and increased LoS have also been shown in older studies [16-18]. In our study, following the introduction of gPCC, both physicians and nurses assessed all patients thoroughly, using their narrative accompanied by self-rating instruments to support understanding. This could probably explain the high correlation between predicted and actual LoS in this group. The patients who stayed longer than predicted did so due to nausea/vomiting or a need for blood transfusion. A study on predicting LoS found that a need for blood transfusion was the most important predictor of prolonged stay and difficult to identify in advance [19]. The need to develop patient assessment has been recognized by other researchers, and recently one of the leading fast-track researchers stated that preoperative assessments should include more details of the patient as a person [20]. In an ongoing study of planned surgical activity, researchers found that during a period of 5 years, around 1,500 patients had planned operations cancelled due to incomplete preoperative preparations [21]. Such mistakes are not only bad for the patients but also costly and disturbing for the clinic.

In our study, patients in the control group were given individual information and they were all informed that the LoS should be 4–5 days. This was not discussed further or related to the individual patient’s circumstances, and they were expected to plan on their own for their rehabilitation/recovery after discharge. To what extent they had actually made a plan and what it included were not evaluated at the pre-admission visit, and this was a clear reason for having an extended hospital stay. Some patients viewed the predicted LoS as unrealistic, and a few of them assumed that the hospital would be responsible for providing care for as long as they needed. This misconception was often not discovered until after the surgery, requiring the ward nurses to involve the community service by sending a notification and booking a discharge planning conference. When this happened, hospital stays were extended by several days and in some cases up to a week. In the gPCC intervention group, the nurses discussed the discharge with the patient at the pre-admission visit and helped the patients understand what they needed to prepare for their return home. The pre-admission visit resulted in a written healthcare plan which was agreed by the patient and, if present, their relative.

The patients’ physical independence before surgery was similar in both groups and most of them were completely independent in both personal ADL and instrumental FRS. At discharge, there was a small difference in personal ADL in favour of the control group. However, after 3 months, the difference was in favour of the gPCC group but neither was significant and both were too small to draw any conclusion.

### Study limitations

Interventions such as those described here are difficult to perform since they involve a large group of staff and affect well-established cultures and routines in the hospital. In order to increase the chances of success, the intervention was planned jointly by patient representatives, physicians, nurses, physiotherapists and occupational therapists in collaboration with the research team. The intention was to establish a working consensus in order to facilitate and safeguard the implementation of gPCC in the designated hospital clinics. The implementation was expected to be difficult, which was confirmed in a pre-intervention study of the resistance to change among the staff [22]. Because of this, a study nurse was recruited to supervise the implementation process and to support staff when needed. During the intervention, recruitment stopped for several months, first because of the summer closedown and also because a shortage of nurses. However, the interruptions were not found to lead to any bias in any of the measured variables (Table 1).

The study was carried out using a quasi-experimental, prospective design in which consecutive patients in an intervention group were compared with patients in a control group representing the usual care. This design was used primarily to avoid difficulties for staff having to work within two care systems simultaneously. A disadvantage of this design is that it precludes evaluation of the true effects of an intervention, for instance, to discover whether the between-group differences are due to the intervention or to other unknown factors. Although random assignment was not applied, the control and intervention groups were comparable at baseline with respect to a large number of clinical and sociodemographic variables. Finally, there was an ongoing project aimed at reducing the length of hospital stay for another patient group at the county hospital, which may have influenced the results in that group.

## Conclusion

Our findings suggest that the gPCC approach was suitable for planned orthopaedic surgery. Patients’ co-morbidities, resources and motivation were found to be equally important factors in planned orthopaedic surgical activity, as had previously been seen in acute orthopaedic care. Despite the resistance to change among the staff, and lack of enthusiasm for following the protocol, a significant difference in LoS was observed between the two groups with maintained health-related quality of life. The result could be interpreted as meaning that the gPCC model was strong enough to overcome such resistance.
